# Replacing a Century Old Technique – Modern Spectroscopy Can Supplant Gram Staining

**DOI:** 10.1038/s41598-017-02212-2

**Published:** 2017-06-19

**Authors:** Shirly Berezin, Yaron Aviv, Hagit Aviv, Elad Goldberg, Yaakov R. Tischler

**Affiliations:** 1 Bar-Ilan Institute for Nanotechnology and Advanced Materials, Bar-Ilan University, Ramat Gan, Israel; 2 Department of Physics, Bar-Ilan University, Ramat Gan, Israel; 3 Department of Chemistry, Bar-Ilan University, Ramat Gan, Israel; 4 Rabin Medical Center, Beilinson Campus, Department of Internal Medicine, Ramat Gan, Israel

## Abstract

Rapid and accurate Gram differentiation is paramount as the first step of pathogen identification and antibiotics administration. However, the current method requires additional reagents, is time-consuming, and is operator dependent. Here we show the principle of tip enhanced Raman spectroscopy (TERS) can differentiate between Gram negative and positive species, by detecting the changes in tip-enhancement in the Raman scattering from the bacteria’s lipid-bilayer membrane, which specifically enhances Gram negative bacteria.

## Introduction

The preliminary analysis of bacterial clinical samples is Gram stain differentiation and has been so for over a century^1^. Currently, initial antimicrobial treatment is administered according to direct microscopic morphology and Gram staining, hours ahead of final biochemical identification of bacteria in culture^2^. Although Gram staining diagnostics can be highly specific, its sensitivity to detect the presence of bacteria is relatively low, requires additional reagents, is time consuming, and can depend on operator technique^3, 4^. Few attempts have been made to improve the sensitivity of this method^5, 6^. Previous efforts to explore spectroscopy as a tool to replace Gram staining have yielded mixed results^7–9^. These methods required complicated procedures and provided relatively low specificity.

Raman spectroscopy is a branch of vibrational spectroscopy that uses the spectrum of inelastically scattered light for interpretation and highly sensitive structural identification of chemicals based on their unique vibrational fingerprints. Raman spectroscopy is an invaluable analytical tool for monitoring changes in molecular bond structure^10^.

Surface-enhanced Raman scattering (SERS) can provide strongly increased Raman signals from molecules which have been deposited onto nanometer sized metallic structures and/or nanostructured metallic surfaces. When the incident laser light in an experimental set-up strikes the metallic surface, the surface plasmons associated with the electronic resonances of the metal localize the electric field on to the adjacent molecules, thereby greatly increasing their efficiency for Raman scattering^11^. Several reports were published on bacterial identification using the SERS spectroscopic technique^12, 13^. In Tip-enhanced Raman spectroscopy (TERS), SERS-like enhancement is produced when a metallized AFM probe amplifies local electromagnetic field that is experienced by the molecules underneath the probe. In addition to the enhancement, TERS can provide Raman spectra with nanoscale spatial resolution^14, 15^. In TERS, the local field enhancement is enabled when the incident laser light strikes the nanosize tip-apex^16^. TERS on bacteria^17–19^ and for other biochemical applications was reported before^20, 21^.

**Figure 1:**
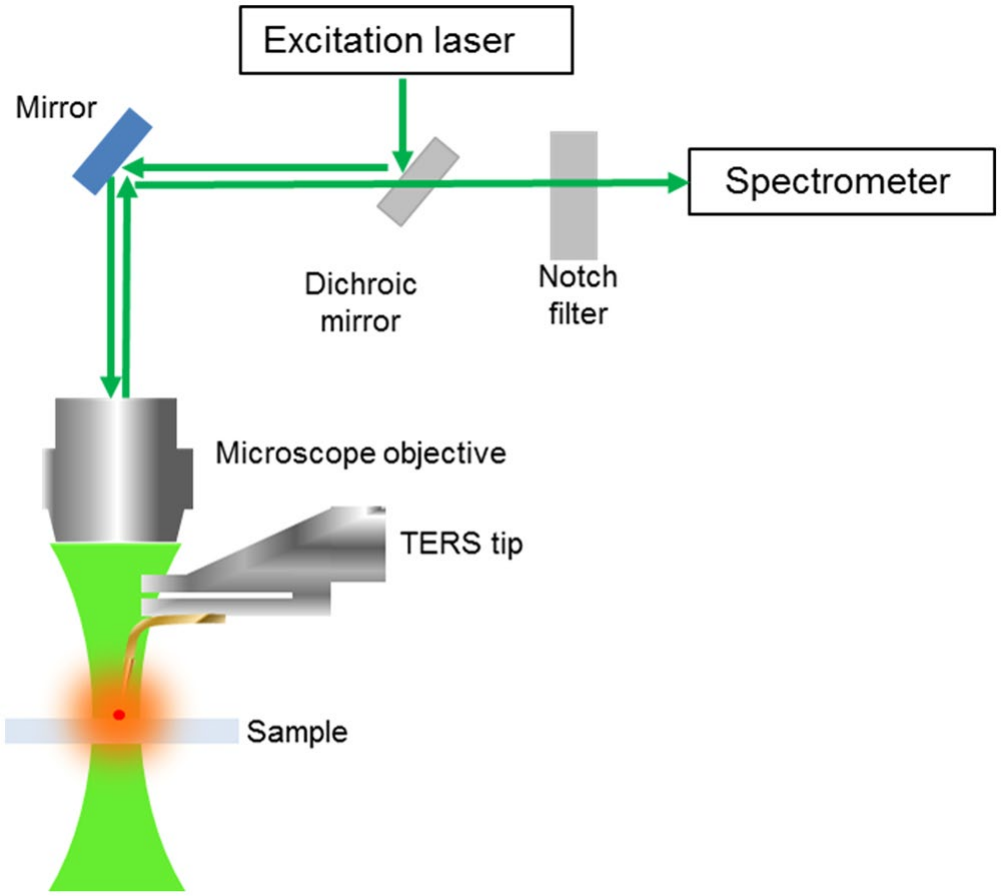
Schematic of the optical set-up for generating TERS.

A schematic of a TERS optical set-up and the principle of the technique are shown in Fig. 1. The excitation laser is reflected by a dichroic mirror towards the microscope objective and then focused on to the sample. The Raman scattering signal is collected by the same objective and then passed through the dichroic mirror and notch filter to the spectrometer, while the laser excitation is filtered out. A sharp metal STM probe or metal-coated AFM tip is positioned under the objective in the focus of the laser. The exact position of the probe is adjusted in order for the laser to excite a plasmonic “hot-spot” of the metallic coating, denoted in Fig. 1 by the red spot in the focus of the excitation. When the laser excites the “hot-spot” in contact or in the near field of the surface of the sample, the electromagnetic field confined to the tip-apex is greatly enhanced due to a combination of localized surface plasmon resonance and lightning rod effects^16^. This increase in electromagnetic field enhances the Raman signal from the molecules in the vicinity of the tip-apex and up to a depth of about 20 nm, depending on the excitation conditions^22^.

**Figure 2:**
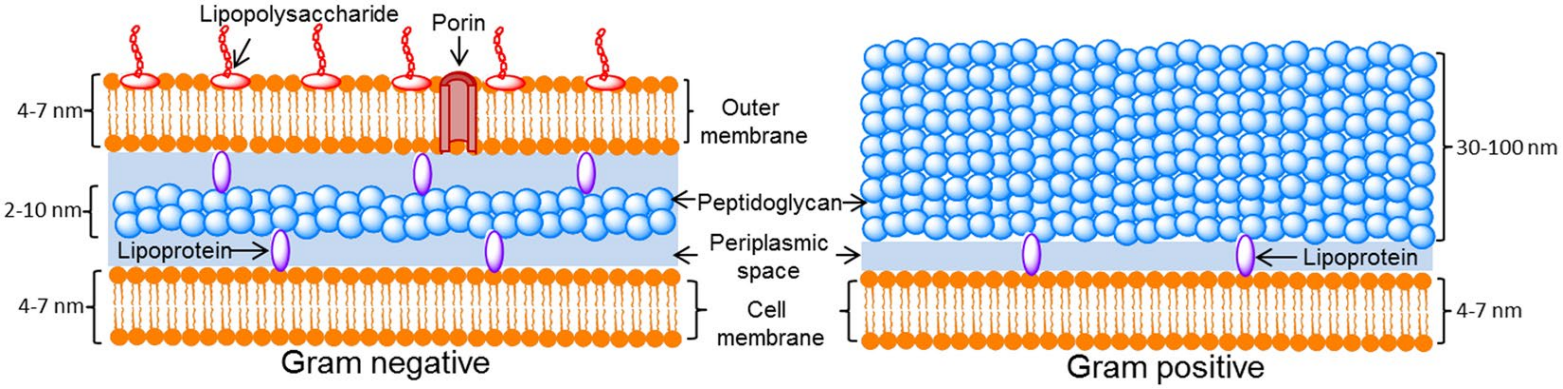
Cell wall structure for Gram negative and positive bacteria.

Figure 2 presents the well-established structural differences between Gram negative and Gram positive bacteria^23^. The cell wall of Gram negative bacteria consists of a thin layer of peptidoglycan (~5 nm) in the periplasmic space between the cell membrane and the outer membrane. The outer membrane contains lipopolysaccharides on its outer surface and channels such as Porins to facilitate non-mediated transport. The structure of Gram positive bacteria consists of a single lipid membrane surrounded by a cell wall composed of a thicker layer of peptidoglycan (30–100 nm)^23^. Lipoproteins interlaced within this structure possess various functions; they bind the membrane via hydrophobic bonds at one edge and the peptidoglycan layer via hydrogen bonds on the other edge^24^.

All cell membranes, including bacterial membranes, are composed of lipid-bilayers. The thickness of the membrane is 4–7 nm and varies with humidity and temperature^25^. The primary role of the lipid-bilayers is to isolate the homeostatic interior cytoplasm from the outside environment^26^. This spontaneously organized bacterial organ has relatively strong Raman shifts in the 2800–3000 cm^−1^ region that are attributed to symmetric and antisymmetric methylene stretching vibrations^27, 28^.

Here we present a novel reagent-free spectroscopic approach for the differentiation between Gram negative and Gram positive bacterial species based on TERS that is faster, simpler, and more accurate than Gram staining. The differentiation relies on the comparison between the Raman scattering intensity from the membrane lipid-bilayers when the TERS tip is out of contact, “Tip-out”, to the signal intensity when the tip is in contact with the sample, “Tip-in”. For Tip-out, TERS cannot be generated on the sample because the tip-apex is outside of the near-field of the sample surface. For Tip-in, the Raman signal from the top layer of the sample which is in contact with the tip can be enhanced. If the top layer contains lipid-bilayers, as is the case for Gram negative bacteria, their Raman signal should be stronger than in the case of Tip-out.

## Results and Discussion

**Figure 3:**
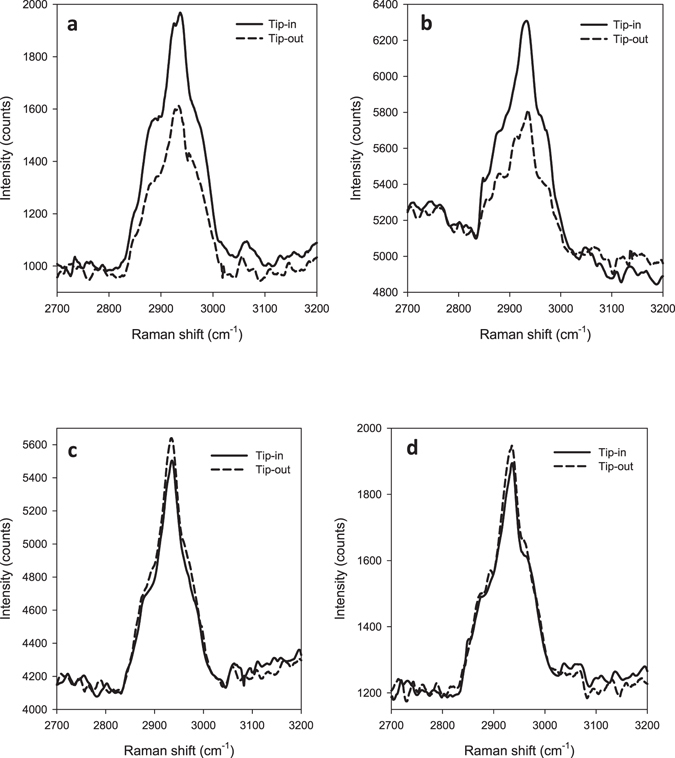
A comparison between Tip-in (solid lines) and Tip-out (dashed lines) for Gram negative and Gram positive bacteria samples. For Gram negative bacteria: (**a**) *Escherichia coli* and (**b**) *Pseudomonas aeruginosa* were measured. For Gram positive bacteria: (**c**) *Bacillus subtilis* and (**d**) *Staphylococcus aureus* were measured.

To investigate our approach, four different bacterial species were examined. Specimens were selected according to Gram staining status. For Gram negative bacteria: *Escherichia coli* and *Pseudomonas aeruginosa* were measured, and for Gram positive bacteria: *Bacillus subtilis* and *Staphylococcus aureus* were measured. Broad spectral range of 1070–3100 cm^−1^ was taken for the four bacterial species and is presented in Figure S1. Figure S1 shows that spectra of different bacterial species vary in all regions except for the membrane’s Raman shift region at 2800–3000 cm^−1^. Since the structural differences between Gram negative and Gram positive bacteria rely namely on the lipid bilayers membrane, which can be found only on the surface of Gram negative bacteria, we focus on this region. Figure 3 shows the Raman spectra in the spectral range of 2800–3000 cm^−1^ methylene stretching vibrations and indicates the differences in the enhancement effect between Gram negative bacteria: *Escherichia coli* (a) and *Pseudomonas aeruginosa* (b) and Gram positive bacteria: *Bacillus subtilis* (c) and *Staphylococcus aureus* (d). For Gram negative bacteria, TERS enhancement is observed, with the signal intensity for Tip-in being more than 60% higher than for Tip-out. For Gram positive bacteria, no enhancement is observed. In fact, a slight reduction in the scattering intensity of approximately 5% is measured due to the tip obstructing the laser excitation and light collection path.

These results demonstrate that the TERS tip enhances the Raman scattering from the methylene stretching vibrations of the Gram negative bacterial cell membrane. We can surmise that the outer membrane is certainly enhanced and probably the inner membrane as well, since both membranes are within the depth of the TERS effect, where plasmonic resonance occurs. In Gram positive species, the lack of an outer membrane and a thicker peptidoglycan layer prevent resonant plasmonic Raman scattering from the membrane, hence no enhancement is observed in their Raman spectra. It is notable that in some measurements, for example in Fig. 3b, the background spectra are tilted. This tilt is attributed to the presence of water molecules within the bacteria or adsorbed to their surface^28^ and has no effect on the bacterial signal. This background is not specimen related and disappears after a few seconds of laser exposure probably due to water evaporation. The intensity of the Raman shifts for the membrane remain the same throughout this evaporation process, and as a consequence the TERS enhancement of the Raman shifts stayed constant for Gram negative bacteria species when comparing Tip-in to Tip-out measurements as expected. Even though the thick peptidoglycan layer of Gram positive species prevents the enhancement in the membrane’s Raman shift, we expect to achieve enhancement in different regions that are assigned to the peptidoglycan layer. For comparison between Tip-in and Tip-out spectra of a broad spectral range, we chose the species that provided cleaner and more detailed spectra, *Pseudomonas aeruginosa* (Figure S2) was chosen for Gram negative and *Bacillus subtilis* (Figure S3) for Gram positive. Figure S2 presents enhancement only for the membrane’s Raman shift region as expected for Gram negative bacterial species. In Figure S3, no enhancement is found for the membrane’s Raman shift, however, the shifts at 1132, 1250, 1303, 1458, and 1578 cm^−1^ are enhanced in the Tip-in spectrum. These shifts are assigned to the polypeptides and the carbohydrates of the peptidoglycan layer^29–31^. The region of 1070–1650 cm^−1^ concludes many other Raman shifts that belong to amino acids^32^ and DNA bases^33^, thus different spectrum is observed for every bacterial species. However, the membrane’s Raman shift is strong and found in all species at the same region, and therefore is proposed as a method for Gram differentiation.

TERS enhances the Raman signal by several orders of magnitude^16^, but since it is localized to a small fraction of the focal volume, it is important to consider the overall increase in the Raman signal. The localized enhancement can be calculated if the spot size of the laser is known as well as the tip-apex radius^22^. In our case, the signal intensity of Tip-in spectra was at least 60% higher than that of Tip-out, from which we derived that a maximal enhancement factor of 10^4^–10^5^ is manifest.

Our results demonstrate a promising method for bacterial differentiation using the TERS principle, wherein the known structural differences between bacterial species are employed in order to differentiate them by comparing Tip-in and Tip-out Raman scattering spectra. Successful differentiation requires no additional reagents and the results are achieved within minutes by applying a fast spectroscopic measurement. Since empirical antibiotic treatment relies heavily on the initial identification of Gram staining, our method can be further developed into a rapid clinical assessment tool. Bacterial differentiation via this innovative use of TERS is a pioneering endeavor in Raman based structural investigation and surface analysis of biological samples.

## Methods

### Materials

Bacteria species: *Escherichia coli, Pseudomonas aeruginosa, Bacillus subtilis*, and *Staphylococcus aureus* were purchased from Biological Industries, Israel. Liquid microbial growth medium, LB Broth, and Phosphate Buffered Saline (PBS) solution were purchased from Sigma-Aldrich, Israel.

### Sample preparation

Agarose cultures are known for their high fluorescence that leads to low signal-to-noise ratio^34^. Therefore, a liquid medium was chosen for growing the bacteria. A sample of each bacterial species was shaken at 37 °C in a test tube with 10 mL of liquid LB for 24 h. Bacteria were precipitated by centrifugation at 6000 rpm for 10 min. After careful removal of the supernatant, LB residues were washed away using 20 mL of PBS solution, employing vigorous mixing, followed by further centrifugation and elimination of the supernatant. Thus, only “washed” bacteria remained. The treated specimens were then carefully spread on a microscope slide for optical measurements. For the broad spectral range, commercial silicon wafer was used in order to avoid the glass high background.

### Optical set-up

TERS measurements were performed using a commercial scanning probe microscope (SPM) system Hydra Multi View 4000 from Nanonics Imaging in the upright microscope configuration. The light path was free-space coupled to a LabRam HR Micro-Raman spectrometer from Horiba Jobin Yevon. The Raman signal was collected in reflection mode through a 50X long working distance objective (N.A. = 0.45, W.D. = 17 mm). The TERS tips contained a 100 nm diameter gold nanoparticle at the apex, also from Nanonics Imaging.

### Hot-spot calibration

**Figure 4:**
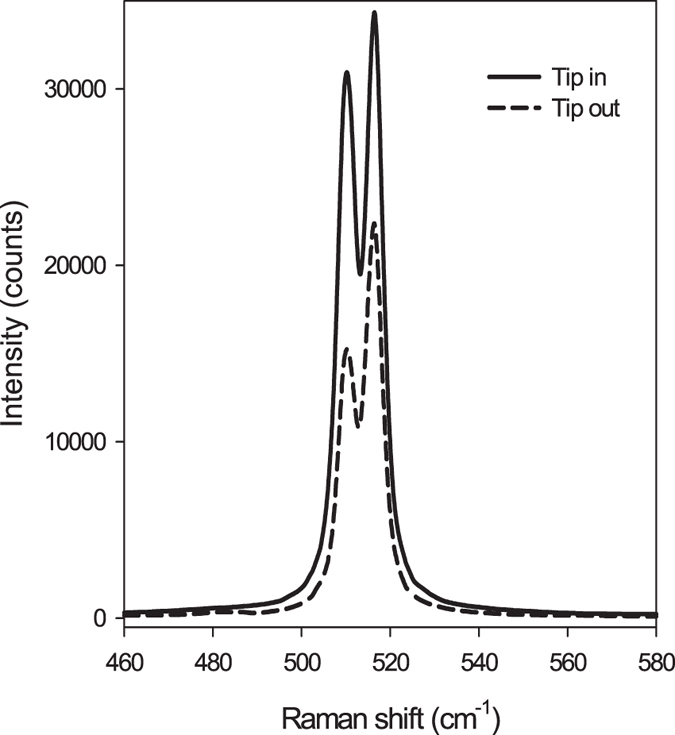
A comparison between Tip-in (solid line) and Tip-out (dashed line) for silicon wafer containing a layer of strained silicon.

Strained silicon wafers are preferred for initial localization of the TERS tip in the “hot-spot” due to high intensity Raman signal. The strong silicon Raman signal enables instantaneous detection of changes in the signal intensity^35, 36^. For our calibration process, a silicon wafer with a thin strained silicon layer at the surface was used (provided by Nanonics Imaging). The Raman shift for strained silicon is at 514 cm^−1^ while the Raman shift for regular silicon is at 520 cm^−1^. When the TERS tip is in contact with the wafer, the intensity ratio between the two Raman peaks changes dramatically. That is, for Tip-in the strained silicon peak increases more significantly than the regular silicon Raman peak, because of the proximity of the strained layer to the TERS tip relative to the bulk of the wafer, as shown in Fig. 4.

In the calibration set-up noted above, the regular silicon Raman shift is also enhanced because regular silicon is also present within the depth of the TERS effect. We confirmed the stability of the laser excitation and the tip location in the “hot-spot” by receiving TERS effect from Gram negative bacteria species before and after every Gram positive bacteria measurement.

### Measurement parameters

Each one of the four bacterial species was grown and spread on at least 8 substrates. Every substrate was measured in at least 5 different spots. The excitation laser in the set-up had a wavelength of 532 nm, and the power used was 6 mW. The acquisition time for each spectrum was 20 sec. For the measurements, a grating with 600 g/mm (2700–3200 cm^−1^) was used.

### Enhancement factor calculations

To determine the localized enhancement factor by calculations, the spot size of the laser as well as the tip-apex radius of the above set-up should be specified. The 532 nm laser spot size diameter is about 2 µm and the penetration depth into the bacterial sample can be calculated^37^ as presented in equation (1):

1 $$L=\frac{2n\lambda }{N{A}^{2}}=\frac{2\ast 1.5\ast 0.532}{{0.45}^{2}}\,\mu m=7.8\,\mu m$$

Where, L is half the focal depth, n is the refractive index of the bacteria^38^, λ is the laser wavelength, and NA is the numerical aperture. It is important to mention that this calculation is correct only when the bacteria layer is thick enough to fill the focal depth, like in our case.

The diameter of the gold particle at the tip apex is 100 nm, and the near-field enhancement occurs to a depth of 20 nm^22, 39–41^ of the bacterial sample. The far-field signal comes from a focal volume of half ellipsoid. The focal volume within the sample is approximated as presented in equation (2):

2 $${V}_{far}=\frac{2\pi }{3}(a\ast b\ast c)=\frac{2\pi }{3}(1\ast 1\ast 7.8)\,\mu {m}^{3}=16.33\,\mu {m}^{3}$$

where a, b, and c are half length of each axis.

While the near-field signal comes from a much smaller volume as calculated in equation (3):

3 $${V}_{near}=\pi {r}^{2}\ast z=\pi \ast {50}^{2}\ast 20\,n{m}^{3}=1.57\ast {10}^{-4}\,\mu {m}^{3}$$

where, r is the radius of the gold particle at the tip-apex and z is the depth of TERS effect.

Since the near-field intensity is 1.6 times higher than the far-field intensity, the contribution from the near-field to the overall enhanced signal is *I*_*near*_ = 0.6 with the far field signal being normalized to *I*_*far*_ = 1.

The enhancement factor when the focal depth is filled with bacterial species can be estimated^42^ by equation (4):

4 $$Enhancement\,factor=\frac{{I}_{near}}{{I}_{far}}\ast \frac{{V}_{far}}{{V}_{near}}=62408$$

This factor is on the same order as the calculated near-field enhancement using the generalized field propagator method^43^.

## Electronic supplementary material

Replacing a Century Old Technique – Modern Spectroscopy Can Supplant Gram Staining
