# Supplementary material for: Replacing a Century Old Technique – Modern Spectroscopy Can Supplant Gram Staining
Source: Sci Rep. 2017 Jun 19;7:3810. doi: 10.1038/s41598-017-02212-2 (PMC5476599; doi:10.1038/s41598-017-02212-2)
Supplement: Supplementary file 1 — Replacing a Century Old Technique – Modern Spectroscopy Can Supplant Gram Staining [file 41598_2017_2212_MOESM1_ESM.pdf]

Replacing a Century Old Technique – Modern Spectroscopy Can Supplant Gram Staining

Shirly Berezin[1,2*], Yaron Aviv[4*], Hagit Aviv[1,3], Elad Goldberg[4], and Yaakov R. Tischler[1,3]

1. Bar-Ilan Institute for Nanotechnology and Advanced Materials, Bar-Ilan University, Israel
2. Department of Physics, Bar-Ilan University, Israel
3. Department of Chemistry, Bar-Ilan University, Israel
4. Rabin Medical Center, Beilinson Campus, Department of Internal Medicine, Israel

* These authors contributed equally to this work.

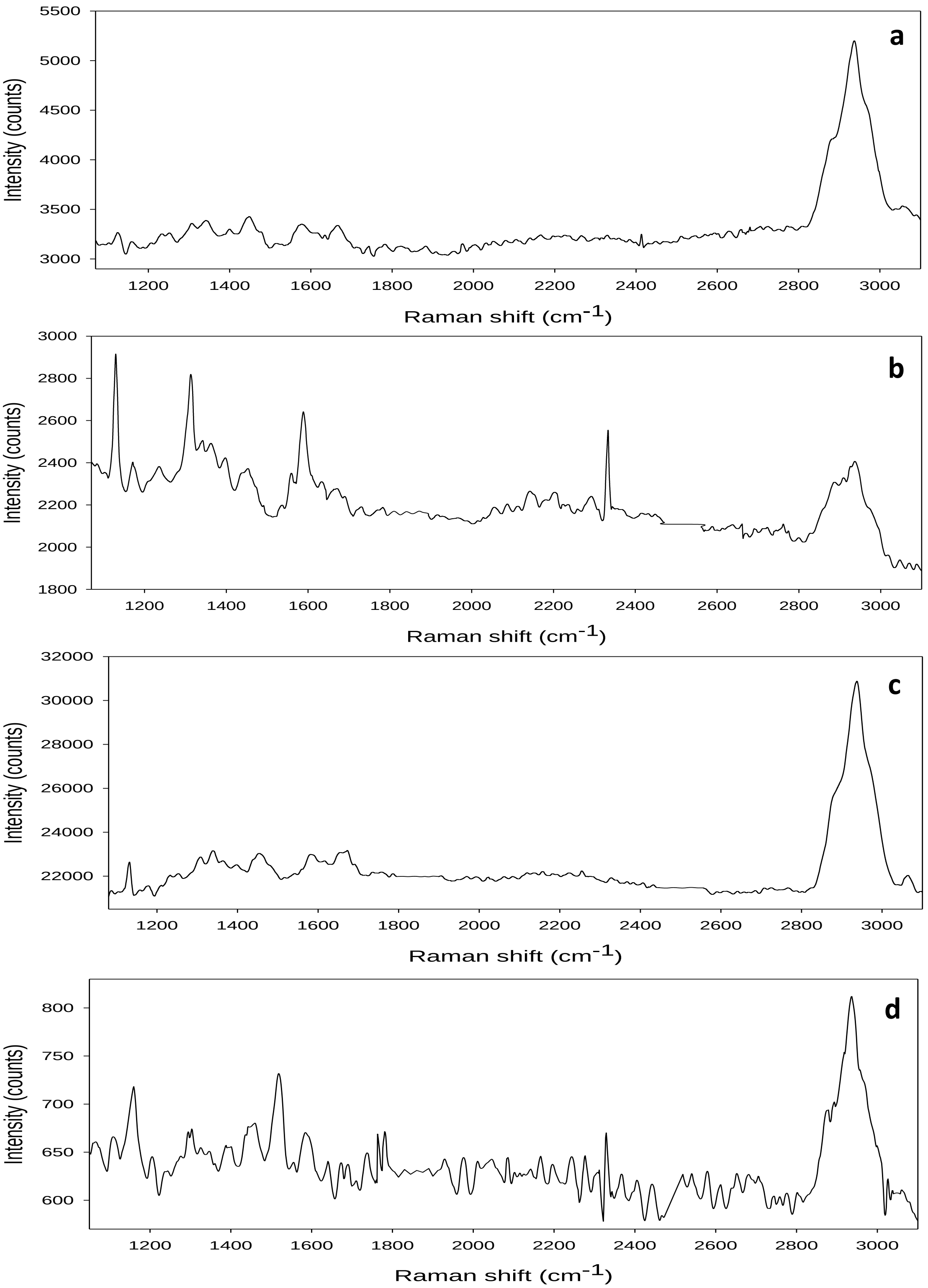

Figure S1: Full spectra for Gram negative and Gram positive bacteria samples. For Gram negative bacteria: (a) *Escherichia coli* and (b) *Pseudomonas aeruginosa* were measured. For Gram positive bacteria: (c) *Bacillus subtilis* and (d) *Staphylococcus aureus* were measured.

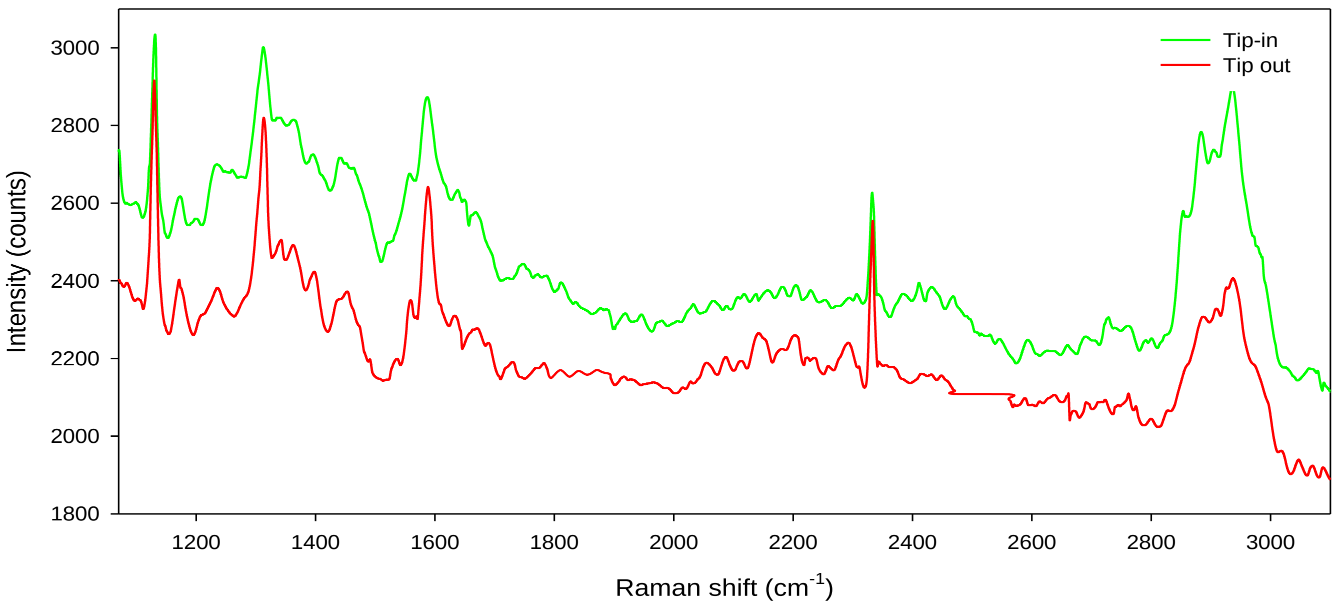

Figure S2: Typical Gram negative spectra for Tip-in (green) and Tip-out (red) measurements. Spectra were taken from *Pseudomonas aeruginosa* bacteria sample.

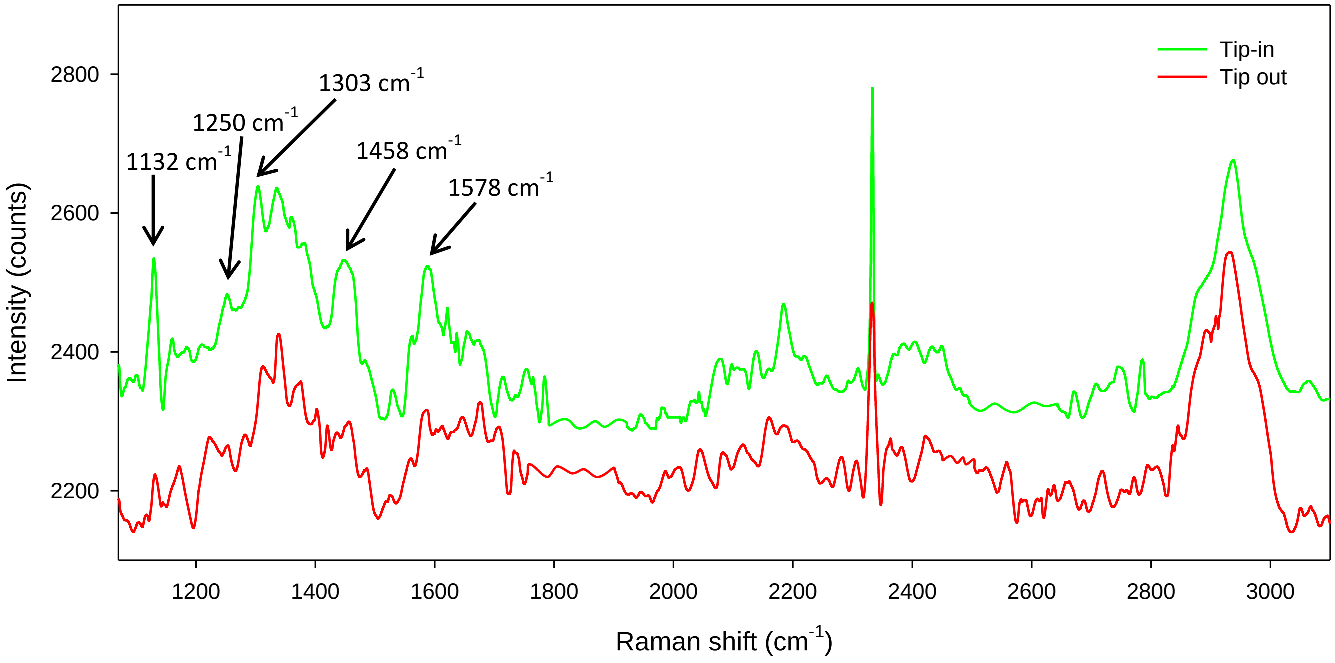

Figure S3: Typical Gram positive spectra for Tip-in (green) and Tip-out (red) measurements. Spectra were taken from *Bacillus subtilis* bacteria sample.
